# Research on chemical characteristics of soil salt crusts with saline groundwater drip-irrigation in the Tarim Desert Highway Shelterbelt

**DOI:** 10.1186/2193-1801-2-S1-S5

**Published:** 2013-12-11

**Authors:** Jianguo Zhang, Xinwen Xu, Jiaqiang Lei, Shengyu Li

**Affiliations:** College of Resources and Environment, Northwest A&F University, Yangling, Shaanxi China; Xinjiang Institute of Ecology and Geography, CAS, Urumqi, Xinjiang China

**Keywords:** Chemical characteristics, Soil salt crusts, Saline groundwater drip-irrigation, Trim Desert Highway Shelterbelt

## Abstract

Soil salt crusts are special layers at soil surface which are widely distributed in the Trim Desert Highway Shelterbelt under drip-irrigation with high salinity groundwater. In order to reveal annual variation of their chemical characteristics, soil salt crusts in shelterbelt of different ages in hinterland of the Taklimakan Desert were sampled. SOM, total salt, inions and pH were analyzed. Following results were obtained. SOM of salt crusts increased with the shelterbelt ages, but increasing trend became lower gradually. Total salt, ions, and pH of salt crusts reduced gradually with the shelterbelt ages. Total salt of salt crusts in shelterbelt of different ages was much higher than shifting sandy land. Ions were higher than shifting sandy land, Cl^-^, Na^+^, and SO_4_^2-^ increased more obvious, then Mg^2+^, K^+^, Ca^2+^ and HCO_3_^-^, CO_3_^2-^ was little and nearly had no change. pH was all alkaline, pH of salt crusts in shelterbelt of 11 years was even lower than shifting sandy land. We can include that the quality of shallow soil (0~30 cm) in the Trim Desert Highway Shelterbelt becomes better gradually.

## Introduction

Soil crusts are widely distributed in land surface, especially prevalent in arid and semiarid regions [1], and are mainly divided into physical crusts and biological crusts [2]. As we know, physical soil crusts are a thin compacted layers formed at the soil surface by the combined action of raindrop impacting, splashing and physicochemical dispersion of the finer particulate matter blocking the soil porosities, or by the sedimentation action of finer particles carried by water current or still water on the soil surfaces [3]. Biological soil crusts commonly result from the development of communities of micro-organisms on soil surface [4–6]. Soil salt crusts are special layers at the soil surface which are mainly formed by soluble salt crystallizing soil particles. Soil salt crusts are different from physical crusts or biological crusts; some scholar thought that it should be classified as chemical crusts individually in the soil crusts classification system [7]. Salt crusts have high salt content, higher hardness and strong resistance to wind erosion [8, 9]. Under natural conditions at the shallow groundwater level regions, water and soluble salt move upwards to soil surface because of soil capillarity action, water evaporates and loses, but the salt accumulates and crystals at the soil surface and forms soil salt crusts. Some scholars made related research on soil salt crusts formed under natural conditions [8, 9], but little studies on soil salt crusts formed under salinity water irrigation were reported [7, 10], and only confined to the relationship between soil salt crusts distribution and irrigation schedule.

Mobile dunes on both sides of the highway are fixed gradually after the construction of the Trim Desert Highway Shelterbelt. As a result of drip-irrigation with local high salinity groundwater (2.8~29.7 g/L), soil salt crusts develop extensively in the shelterbelt [7, 10]. Under salinity water drip-irrigation, salt accumulates at soil surface, and salt content of distribution layers of trees roots is lower, so salt-injuries don't happen. But, unexpected precipitations can leach salt of soil salt crusts downwards, if soil salt contents of distribution layers of roots exceeds trees' salt-tolerance threshold, plants physiological drought might happen, even results in plants death. Unexpected strong precipitations happened in the summer of 2003 & 2005, which caused a lot *Calligonum* L. died and caused certain bad influence on protection of the shelterbelt.

The objective of this study was to reveal the chemical characteristics of soil salt crusts under high salinity groundwater drip-irrigation, and this study has important practical application value and theoretical meaning for analyzing and resolving salt-injuries of precipitations to the shelterbelt and promoting sustainable development of the shelterbelt.

## Materials and methods

### The study area

**Figure 1:**
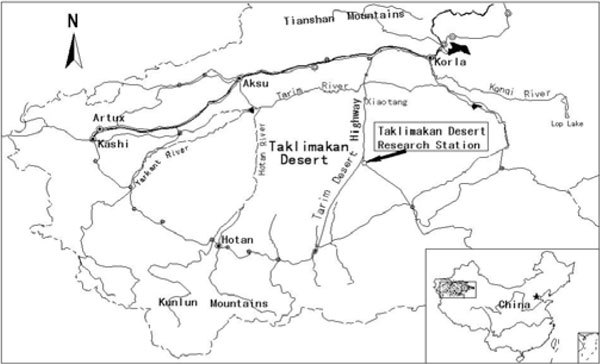
**Map showing the study area**.

The study was conducted at the Taklimakan Desert Research Station/Tazhong Botanical Garden, Chinese Academy of Sciences, in the hinterland of the Taklimakan Desert in Xinjiang Uighur Autonomous Region (approximately 39º01´ N, 83º36´ E, 1 100 m a.s.l.) (Figure 1). The climatic characteristics of study area are as follows: (1) The annual mean air temperature is 12.4°C, with December the coldest month averaging -8.1°C, July the warmest month averaging 28.2°C; (2) The climate is extremely arid, annual average precipitation is approximately 24.6 mm, annual average relative humidity is 29.4%, number of low humidity days (≤30%) is 246.6 days, and annual mean potential evaporation is 3 638.6 mm. (3) Blown sand disasters are serious, the annual average wind speed is 2.5 m/s, maximum instantaneous wind speed is 20.0 m/s, and the sand-shifting wind occurs more than 130 days per year.

Since 1997, to protect the security of the Trim Desert Highway, the shelterbelt ecological system was experimentally established and succeeded in the hinterland of the Taklimakan Desert on both sides of the highway, and was popularized along the Trim Desert Highway in 2005. The species are mainly highly stress-resistant shrubs with excellent windbreak and sand fixation properties, such as *Calligonum* L., *Tamarix* L., *Haloxylon bunge* and etc. All of the shelterbelt trees are drip-irrigated with regional high salinity groundwater, and soil salt crusts are formed and widely distributed in it [7].

In the hinterland of Taklimakan Desert, natural vegetation is rare without a few drought-resistant shrubs (*Tamarix ramosissima* and *Calligonum leucocladum*) growing in lowlands between dune-chains, the coverage is very low; ground landscape is mainly high mobile dunes and large complex dune chains. The soil is mainly shifting aeolian sandy soil which has little nutrients, the salt content is 1.26~1.63 g/kg and pH is 8~9 (Table 1). The depth of groundwater at lowlands between dune-chains is 3~5 m, the mineralization is 4.0~ 4.8 g/L, ions composition are mainly Cl^-^, SO_4_^2-^, Na^+^ and K^+^. The water quality profile is shown in Table 2.

**Table 1:** Physical and chemical properties of shifting aeolian sandy soil at the study area

pH/1:5	Conductivity/ms/cm	Total salt content/g/kg	Ions composition/g/kg								particle composition/%					
			**CO**_**3**_^**2-**^	**HCO**_**3**_^**-**^	**Cl** ^**-**^	**SO**_**4**_^**2-**^	**Ca** ^**2+**^	**Mg** ^**2+**^	**Na** ^**+**^	**K** ^**+**^	**Clay**	**Silt**	**Very fine sand**	**Fine sand**	**Medium sand**	**Course sand**
8.26	0.437	1.33	0.02	0.106	0.703	0.014	0.096	0.01	0.32	0.061	0.27	12.35	52.05	30.79	2.12	2.42

**Table 2:** Irrigation groundwater quality profile at the study area

pH	Conductivity / ms/cm	Mineralization / g/L	Salt content / g/L	Ion composition/g/L					
				**HCO**_**3**_^**-**^	**Cl** ^**-**^	**SO**_**4**_^**2-**^	**Ca** ^**2+**^	**Mg** ^**2+**^	**K** ^**+**^ **+ Na** ^**+**^
8.13	6.06	4.04	3.912	0.079	1.497	1.005	0.108	0.150	1.073

### Experimental design and layout

In the study area, the shelterbelt is drip-irrigated with high salinity groundwater whose mineralization is 4.04 g/L. The shelterbelt is irrigated once/15d in March, April, May, September and October, once/10d in June, July and August, irrigation quantity is 30 L/m^2^ once, and without irrigation from November to February next year.

In late March 2007, soil salt crusts were sampled in the shelterbelt planted for 11 years, 8 years, 5 years, and 2 years in the hinterland of the Taklimakan Desert, meanwhile, shifting aeolian sandy soils were collected as checks. To obtain organic matter contents, total salt contents, eight ions contents and pH, all samples were air-dried, rushed and passed through 2.0 mm sieve before analysis.

SOM was determined by potassium dichromate method; Total salt was determined by gravimetric analysis; Ca^2+^ and Mg^2+^ were determined by EDTA volumetric method; K^+^ and Na^+^ were determined by flame photometry; SO_4_^2-^ was determined by barium sulfate turbidity; Cl^-^ was determined by silver nitrate titration; CO_3_^2-^ and HCO_3_^-^ were determined by neutralization titration; pH (soil: water = 5:1) was determined by glass-electrodes method [11].

## Results

### SOM analysis

**Figure 2:**
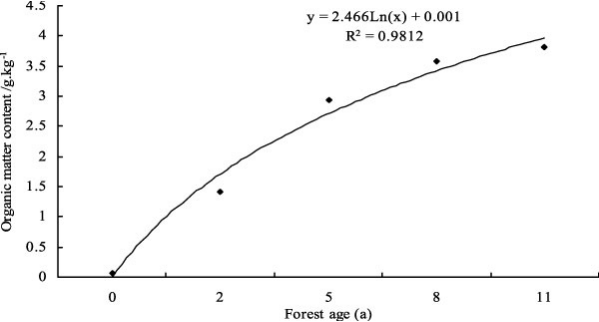
**SOM analysis of soil salt crusts in shelterbelt of different age**.

It is known that SOM can decrease soil bulk density, increase soil porosity, improve soil structure and aeration, improve soil infiltration, soil water and heat conditions, create better conditions for plants growth and development. It is known from Figure 2 that SOM of soil salt crusts increased with forest ages in the shelterbelt drip-irrigated with salinity groundwater. The analysis results showed that the SOM of soil salt crusts in shelterbelt irrigated for 11 years, eight years, five years and two years increased by 18.15 times, 38.49 times, 47.21 times and 50.33 times compared with shifting sandy land. But, it still can be seen from Figure 2 that the increasing trend weakened gradually. At study area, species of shelterbelt are mainly deciduous shrubs, such as *Calligonum* L., *Tamarix* L. and *Haloxylon Bunge*, etc. Litters are accumulated in the shelterbelt, and the increase of SOM is closely related to continuous accumulation and decomposition of litters [12, 13].

### Total salt analysis

**Figure 3:**
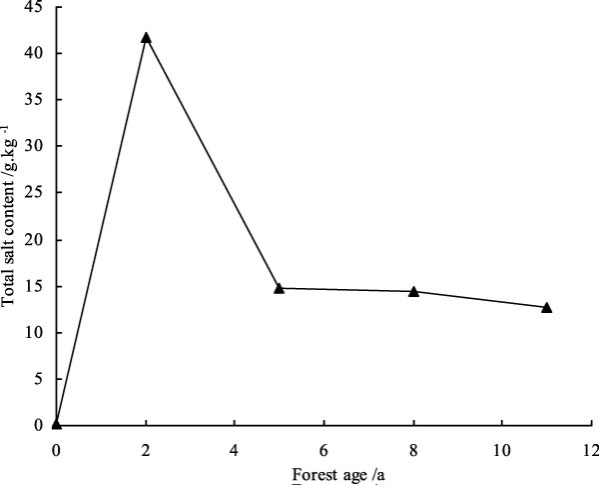
**Analysis of total salt of soil salt crusts in shelterbelt of different age**.

Soluble salt content in soil is one important index of judging the degree of salinization, it is one of important limiting factors to plant growth. Salt-injury or alkali-injury might be happened to plants if soil salt content is excessively high [14, 15]. Mineralization of irrigation water in study area is 4.04 g/L. Figure 3 showed that salt contents of soil salt crusts in shelterbelt with salinity water drip-irrigation change much, and was much higher than shifting sandy land, but reduced gradually with the forest ages. This conclusion is consistent to the conclusions of Zhou's and Xu's [16, 17]. Salt contents of soil salt crusts in shelterbelt irrigated for two years, five years, eight years and 11 years increased 317.15 times, 111.19 times, 108.96 times and 95.65 times than shifting sandy land. Salt contents of soil salt crusts reduced gradually with shelterbelt irrigation years, which are contributed to continuous salt leaching of drip-irrigation [18].

### Ions composition analysis

**Figure 4:**
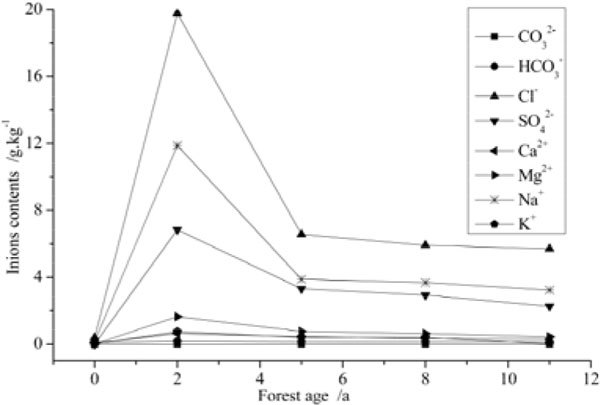
**Analysis of ions contents of salt crusts in shelterbelts of different age**.

Under high salinity groundwater irrigation, salt is brought into soil with irrigation water; ions composition and contents of the soil are affected. In shifting sandy land, soil anions are mainly Cl^-^ and HCO_3_^-^, a spot of SO_4_^2-^ and little CO_3_^2-^; cationics are mainly Na^+^ and Ca^2+^, a spot of Mg^2+^ and K^+^ (Table 1). It can be inferred from Figure 4 that under salinity groundwater drip-irrigation, ions contents of soil salt crusts in shelterbelt irrigated for different years increased at different degree compared with shifting sandy land, but reduced gradually with the shelterbelt ages, the trend was consistent with total salt contents of salt crusts. SO_4_^2-^ content of soil salt crusts in shelterbelt irrigated for different years (two years, five years, eight years and 11 years) increased 157~477 times than shifting sandy land; Cl^-^ increased 7~27 times; HCO_3_^-^ increased 0.31~0.73 times which increased slightly; CO_3_^2-^ content was very few, and nearly had no difference with shifting sandy land; Mg^2+^ increased 43~164 times; Na^+^ increased 9~36 times; Ca^2+^ increased 2.2~5.6 times; and K^+^ increased 5.5~11.1 times. It can be inferred from analysis results that Cl^-^, Na^+^ and SO_4_^2-^ increased most obvious; then Mg^2+^, K^+^, Ca^2+^ and HCO_3_^-^; CO_3_^2-^ content was very few and nearly had no change. Ions composition of soil salt crusts was accordant with irrigation water (Table 2).

### 4 pH analysis

The increase of salt content may result in soil salinization or alkalization. Sodium salt, calcium salt and magnesium salt, formed by soil ions will restrict plants growth and development at different degrees, or even lead to plants death when serious [15]. pH is one of important parameters of soil chemical characteristics, and is one of important indexes of judging soil salinization or alkalization. It can be inferred from the analysis results in Table 3 that pH of soil salt crusts in shelterbelt of different ages (2 years, 5 years, 8 years and 11 years) were all alkaline, and decreased gradually with shelterbelt ages. This trend was consistent with the change of total salt contents and ions composition of soil salt crusts, pH of soil salt crusts in shelterbelt of 11 years was even lower than shifting sandy land.

**Table 3:** pH (water: soil = 5:1) of salt crust in shelterbelt irrigated for different years

Forest age/a	Shifting sandy land	2	5	8	11
pH	8.26	8.86	8.62	8.3	7.93

## Conclusions and discussions

From all the analyses it can be concluded that: (1) in the shelterbelt with high salinity groundwater irrigation, SOM of soil salt crusts increased with shelterbelt ages, but increasing trend weakened gradually; (2) total salt of soil salt crusts was much higher than shifting sandy land, and reduced with shelterbelt ages because of soil salt leaching by drip-irrigation; (3) salt was mainly brought into soil by drip-irrigation with saline water, ions contents of soil salt crusts in shelterbelt were much higher than shifting sandy land, but reduced with shelterbelt ages gradually. Increment amounts of Cl^-^, Na^+^ and SO_4_^2-^ were largest, then Mg^2+^, K^+^, Ca^2+^ and HCO_3_^-^, the content of CO_3_^2-^ was very less and nearly has no change. This change was consistent to ions composition of irrigation water; (4) pH of soil salt crusts in shelterbelt of different ages was all alkaline, but decreased with shelterbelt ages; pH of salt crusts in shelterbelt of 11 years was even lower than shifting sandy land.

Under irrigation with salinity groundwater, salt crusts form at the soil surface which has impact structure and certain thickness. We discovered that it can greatly improve soil resistance to wind erosion and can inhibit soil evaporation effectively. In arid area, soil moisture is one of main limiting factors affecting vegetation growth, development and distribution. It rains very less and evaporates strong in the extreme drought Taklimakan Desert region; excessive evaporation causes a large amount of irrigation water loss ineffectively, which results in harmful effect on growth and development of plants. So the salt crusts can play a role on saving the limited water resource for irrigation. Salt crusts are at the soil surface, and commonly don't influence normal growth and development of the shelterbelt. but unexpected strong precipitations can leach salt of salt crusts downwards, plants normal growth will be affected even die if the leaching depth arrives at main distribution layers of plants roots [19]. Therefore, arrangement for irrigation system reasonably and preventing the occurrence of salt-injuries is one of the issues to maintain the stability of the desert highway shelterbelt.

Research on chemical characteristics of soil salt crusts under salinity groundwater drip-irrigation not only has important practical application value and theoretical value in analyzing and solving precipitation salt-injuries on shelterbelt, promoting the sustainable development of the shelterbelt, but also have reference meaning in preventing the occurrence of regional salinization and guiding rational use of water resource in salinity water irrigation region.

In the research field of salt crusts formation, evolution process and mechanism, and their role in ecosystems are not systematic enough [20]. Salt crusts formation and development process is affected by irrigation water quality, groundwater depth, soil texture, vegetation, climate conditions, etc; soil eco-hydrological process under the influence of salt crusts and their effect on plants growth and development, etc, these aspects are worthy of further research and discussion.
